# The Employment of the Waiting Years

**Published:** 1918-04-27

**Authors:** 


*To the Editor of* THE HOSPITAL.

### The Employment of the Waiting Years.

*To the Editor of* THE HOSPITAL.

SIR,—The article on "The Employment of the Waiting Years" in THE HOSPITAL of April 20 has interested me exceedingly as I spend my life in wrestling with the results of such years!

In thinking about the matter during the three years in which I have been engaged in the formal education of probationers, it has often occurred to me that it is such a pity that the head-mistresses of high schools and higher elementary schools could not be approached with a view to co-operation with matrons of training-schools in advising girls on the best way in which to spend "the waiting years," and I have been on the watch for some opportunity to bring me into touch with educational authorities.

Strangely enough, the opportunity seems at last to have presented itself. I have been told that head-mistresses will probably eagerly welcome any suggestions which will tend to keep the girls longer at school and help them to shape their careers wisely. The restlessness of the times is much affecting schools. I am told that a continual topic of conversation in the mistresses' common room to-day is "How can I advise So-and-So"? The girls are indefinite and the parents more indefinite still as to what they want.

My idea is that matrons and teaching sisters should visit suitable schools and speak to the higher forms in a friendly, informal way about nursing as a life occupation. They could surely gather up enough material from their own and their friends' experiences to fire the enthusiasm of the girls for work among the sick-poor. The average girl in her teens is brimming over with kindly sympathy if approached in the right way. It would add to the impression if the speaker wore becoming uniform well put on, and looked as if nursing had really been an enjoyable occupation. The dragon sister alluded to in recent issues would not be of much use!

Then the possibility of a vocation might be spoken of and it might be added that, as a wise man once said: "A need for service and a power to give that service *is* a vocation." This would naturally lead up to the necessary preparation. It could be pointed out how a mistake in calculating fractions in measuring hypodermic injections might mean the loss of a precious life, how decimals must be clearly understood, and how essential are clear writing and good spelling for reports. Then they could be urged to learn some biology as an introduction to anatomy and physiology, and to get a clear grasp of at least the elementary principles of physics and chemistry to prepare the way for hygiene. The value of Latin and modern languages could also be explained; in fact, how nothing in the shape of knowledge comes amiss. It would be too much to expect girls of fifteen or sixteen to appreciate a liberal education at its true worth, so it would be necessary to dwell on the utilitarian aspect of their subjects. I, myself, had less difficulty than most with a refractory old gentleman because, when he was "good," I read his Vulgate to him, and a delirious patient would take anything when spoken to in the language of his childhood—French. I could multiply instances of the value of a higher education to a nurse, some most comical in character.

Remarks of this kind would soon show the girls that those who are anxious to leave school from the middle or upper fourths are not the kind of girl wanted in the training-schools, and would reconcile them to a longer school life. It could also be pointed out to them how in the upper forms they would get the training in taking notes of lectures—such an insuperable difficulty in the training-schools. A sister who actually teaches nurses once admitted to me that she could only take notes as dictation. It would also show the clever girls that it is not beneath their intellectual dignity to think of nursing as a career. The idea of the College of Nursing with its standardised examinations would probably appeal to clever girls very much.

This would lead up to advice as to how to spend the waiting years. If it were necessary to earn they could be advised to take up some work which would mean the constant use of their brains and teach them business habits, a weak point with most nurses, and they should be advised to attend courses of lectures to keep up the habit of regular study and answering examination papers. Almost any scientific or literary subject would be good, except anatomy, physiology, or nursing. These only lead to confusion of mind and staleness later on. For exactly the same reason I deprecate training of any kind in sick nursing before the general training.

Housework is of little use as practised in the average private house. I find that the best housewives I turn out are those who have never handled, before their training, broom or dish-cloth, as they begin with an innate dislike of anything "nasty," which makes them dainty and thorough workers, though, perhaps, not as quick as some of the slap-dashers who can never distinguish the cloths in the sink-room from one another. Good needlework is a very valuable asset however.

If it is not necessary to earn, a University education, especially in science, domestic economy courses, physical education, kindergarten training are all good ways of filling up the time. Whether obliged to earn or no, all intending nurses should be urged to study social conditions and try to understand conditions of life among the working classes from the point of view of the sympathetic friend, not the reformer, and also to get good teaching in cookery, available practically everywhere in the evening classes at Polytechnics.

A homely suggestion will make life much easier for the probationer nurse if acted upon. That is that she should gather together in the waiting years an ample trousseau of plain, strong clothing calculated to withstand the ravages of the hospital laundry. This will give her more leisure for recreation and study while training. The study of child-life is most important, too, to the aspiring nurse for obvious reasons.

I feel constrained to write to you as the whole question is so serious. The girls who are restlessly clamouring to-day to leave school at fourteen or fifteen are our probationers of to-morrow! It is not a happy outlook for nursing!—I am, Sir, yours faithfully,

A Teaching Sister-in-Charge.

April 21, 1918.

